# Application of PDCA cycle management for postgraduate medical students during the COVID-19 pandemic

**DOI:** 10.1186/s12909-021-02740-6

**Published:** 2021-05-29

**Authors:** Shixian Gu, Aijing Zhang, Gang Huo, Wenqing Yuan, Yan Li, Jiangli Han, Ning Shen

**Affiliations:** 1 Department of Education, Peking University Third Hospital, Beijing, 100191 China; 2 Department of Cardiology, Peking University Third Hospital, Beijing, 100191 China; 3 Department of Pulmonary and Critical Care Medicine, Peking University Third Hospital, Beijing, 100191 China

**Keywords:** Postgraduate medical students, COVID-19, Plan-do-check-act cycle

## Abstract

**Background:**

The COVID-19 outbreak has exerted an enormous impact on various industries worldwide. During this pandemic, clinical teaching hospitals have faced unprecedented challenges regarding the management of postgraduate medical students since postgraduate students in clinical medicine have both student and resident identity characteristics. The purpose of this study was to explore the management effectiveness of Peking University Third Hospital (PUTH) based on PDCA (plan-do-check-act) cycle management and to further develop the medical student management system during the pandemic.

**Methods:**

The methods of document review, questionnaire surveys and interviews were used to continuously improve the management measures for postgraduate medical students during the COVID-19 pandemic by using the PDCA cycle.

**Results:**

Investigations were conducted on the management system, back-to-school arrangements, laboratory management, COVID-19 prevention and control training, online teaching, mentoring, dissertation progress, and emotional state of postgraduate medical students during the COVID-19 pandemic. We found that strengthening public health management knowledge training, increasing infectious-disease-related knowledge training, innovating online teaching methods, improving PDCA management model maps, and formulating improvement programmes are conducive to improving the quality of such management.

**Conclusion:**

Given the difficulties involved in the management of postgraduate medical students during the COVID-19 pandemic, managers need to comprehensively consider and conduct overall planning and use the PDCA management model to improve the management of postgraduate medical students during this period.

**Supplementary Information:**

The online version contains supplementary material available at 10.1186/s12909-021-02740-6.

## Background

Since December 2019, cases of pneumonia from a novel coronavirus (COVID-19) infection have occurred in China and other countries, and the number of cases has continued to increase rapidly, causing widespread global concern. On the evening of January 30, 2020, the World Health Organization (WHO) announced that the new coronavirus outbreak was considered a public health emergency of international concern (PHEIC) [1]. Subsequently, COVID-19 has spread worldwide. On February 28, 2020, the WHO evaluated the global risk of the COVID-19 disease as “very high”, and the European Centres for Disease Control and Prevention raised the European risk level to “moderate to high”. In the early morning of March 12, 2020, Beijing time, the WHO officially characterized the COVID-19 pandemic as a global “pandemic”. When the WHO released this news, more than 110 countries and regions in the world had recorded a total of 118,000 confirmed COVID-19 cases, and more than 4000 people had died. Joint prevention and control are an inevitable requirement for responding to infectious diseases in the context of globalization. The outbreak of the COVID-19 pandemic has exerted an enormous impact on various industries worldwide. Since the beginning of the COVID-19 pandemic, a large number of postgraduates with master’s and doctoral degrees in China have been undergoing standardized training for residents. As a university-affiliated hospital for cultivating future medical talent, Peking University Third Hospital (PUTH) has faced unprecedented challenges regarding the management of postgraduate medical students.

**Fig. 1:**
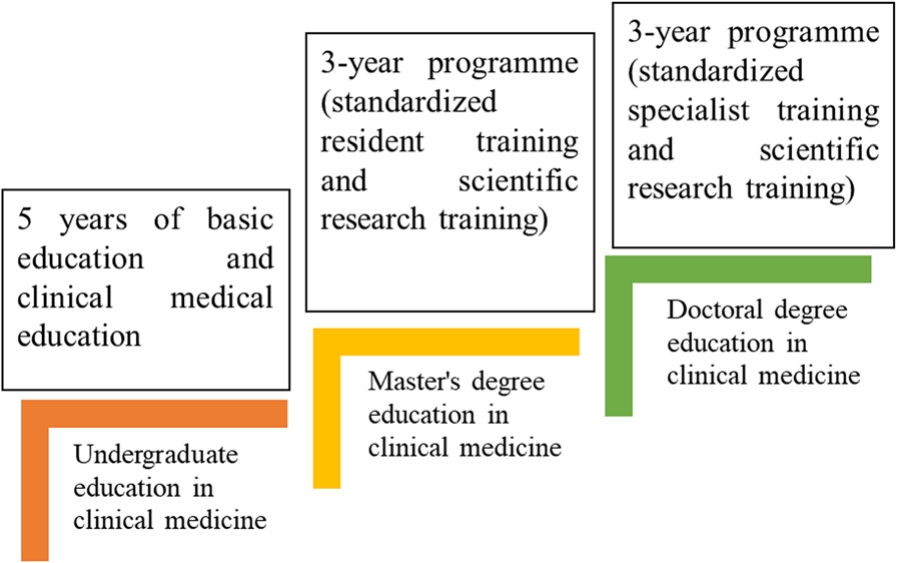
Chart of training mode for postgraduate clinical medical students

In China, high-school graduates can be selected to enter medical school by taking the unified national examination and can earn a bachelor’s degree after completing 5 years of general education and clinical medical education. Graduate students can further apply for a master’s degree in clinical medicine through two pathways: national examination or school recommendation. Those who have achieved outstanding results after 3 years of study can continue to apply for a three-year doctoral degree in clinical medicine, which is called the “3 + 3” continuous training model (Fig. 1). In addition to providing standardized training to residents, a master’s degree in clinical medicine focuses on cultivating professional basic theories, comprehensive abilities, and innovative spirit. In addition to 3 years of standardized training for senior residents, students applying for the clinical doctoral degree must also receive 1 year of scientific research training to achieve the goal of cultivating innovative talent. Because postgraduate students in clinical medicine have both student and resident identity characteristics, student management at medical schools and hospitals has become complicated as well as challenging during the COVID-19 pandemic.

The plan-do-check-act (PDCA) cycle, also known as the “quality loop”, is a general model in management that originated in the 1920s. Walter A. Shewhart proposed the concept of “plan-do-see” (PDS), which was further developed by W. Edwards. Deming, an American quality management expert, into the “plan-do-see-check-act” cycle, also called the “Deming circle”. The Deming circle was introduced to Japan and China in the early 1950s and late 1970s, respectively. It was initially used in total quality management and was subsequently extended to various work areas in numerous industries. Similarly, the application of the Deming circle in the teaching field has also promoted the improvement of teaching quality [2–4]. The PDCA cycle is divided into four phases: plan, do, check, and act. PDCA performance involves the entire management system being in a four-stage cycle of “planning-execution-inspection-processing” at all levels and links, which reflects the internal logic of system operation. The management system of the entire organization constitutes a large cycle, and each phase of PDCA has its own smaller cycle, together forming a large-scale, small-ring, interrelated and mutually restricted scientific cyclic system.

In accordance with the actual situation of PUTH, the PDCA management mode was used to thoroughly explore and analyse the management process and strategy of postgraduate medical students as residents during the pandemic. These management processes and strategies have important practical significance and reference value for ensuring the safe resident training of postgraduate medical students during the prevention and control of pandemics.

## Methods

### Study design

This study was conducted in the context of management measures for postgraduate medical students during the COVID-19 pandemic in PUTH. The scope of management comprised management guidelines, teaching and supervision, and mental health monitoring. A total of 276 clinical professional degree postgraduates in PUTH participated in this study. Document review, questionnaire surveys (See Supplementary File 1) and interviews were used to continuously improve the management measures for postgraduate medical students during the COVID-19 pandemic through the PDCA cycle.

### Data collection

Data was collected over a two-month period after Wuhan was closed. The overall return rate of students to the hospital was collected. COVID-19 prevention and control training and a survey of postgraduate psychological status were conducted.

### Statistical analysis

The data was managed with Excel 2019. SPSS 25.0 was used for statistical analysis. The quantitative data conforming to the normal distribution was described as mean ± standard deviation. The qualitative data was described as number (percentage), and the comparison between groups was performed with the Chi-square test. In all tests, statistical significance was set at two-tailed *P* values less than 0.05.

## Results

### Plan

At the beginning of the COVID-19 pandemic outbreak, little was known about its transmission route, pathogenesis, epidemiological characteristics, etc. Therefore, hospitals needed to develop comprehensive plans for the management of postgraduate medical students that included management guidelines, teaching and supervision, and mental health monitoring.

### Management guidelines

Since the COVID-19 outbreak occurred during the Spring Festival, most postgraduate students had returned to their homes, and some students had travelled abroad. Therefore, arranging for these students to return to the hospital on time to continue participation in their clinical rotation was the first management issue. The period from January to April is a special one for graduate students, as this time is typically used to write dissertations and thesis reviews, complete their graduation thesis defences and obtain employment, which also increases the difficulty of managing postgraduates during pandemic prevention and control.

In addition to clinical training, postgraduate medical students also undertake scientific research. To make further progress in their subject, some students needed to return to school to continue their experiments.

### Teaching and supervision

As a comprehensive tertiary hospital, PUTH has high-risk departments, such as the infectious disease department (including the fever clinic), emergency department, and the pulmonary and critical care medicine department (including the negative pressure ward). Postgraduate medical students needed to rotate through these departments and quickly conduct emergency COVID-19 protection training to reduce the risk of COVID-19 infection.

The average age of the 276 postgraduates in clinical medicine at PUTH is 26 years old (22–32 years old). In the early stage of the COVID-19 pandemic, students may not have fully implemented qualified protective measures due to insufficient knowledge of COVID-19 and inadequate protective materials in the hospital.

### Mental health monitoring

With the increase in the number of new confirmed cases and deaths each day, postgraduate medical students working on the clinical frontline may have experienced negative emotions such as fear, terror, and anxiety, and their parents may have also experienced various anxieties and concerns. Therefore, it was necessary to attend and respond comprehensively to the psychological changes experienced by these students.

Based on the above-mentioned management problems, to ensure zero infections among these students and to promote the steady, orderly development of clinical training and scientific experiments, it was decided that the hospital should immediately establish a COVID-19 pandemic prevention and control team and an emergency management system.

### Do

In the early days of the COVID-19 outbreak, the hospital set up pandemic prevention and control leading and working groups as soon as possible to comprehensively deploy and implement various pandemic prevention and control tasks and teaching work. In accordance with the “Peking University Notice on Postponing the Start Time of the Spring Semester of 2020” and other documents issued by Peking University Health Science Centre (PKUHSC), the hospital issued the “Work Plan on Student Management during the Prevention and Control of COVID-19 in PUTH”. Through the strict management of temporary student dormitories, attention to students’ psychological crises, and establishment of emergency treatment procedures for students with fever, the hospital aimed to do its best to manage these students during the COVID-19 pandemic.

The postgraduate supervisor is the first person responsible for postgraduate training. PKUHSC required the supervisor to provide remote guidance (online and offline) to the graduate student and keep records of such guidance. It was recommended that supervisors pay more attention to the physical and mental health of graduate students, clinical and scientific research work, and pandemic prevention and control.

### Management guidelines

#### Develop principles for returning and after returning to school

**Fig. 2:**
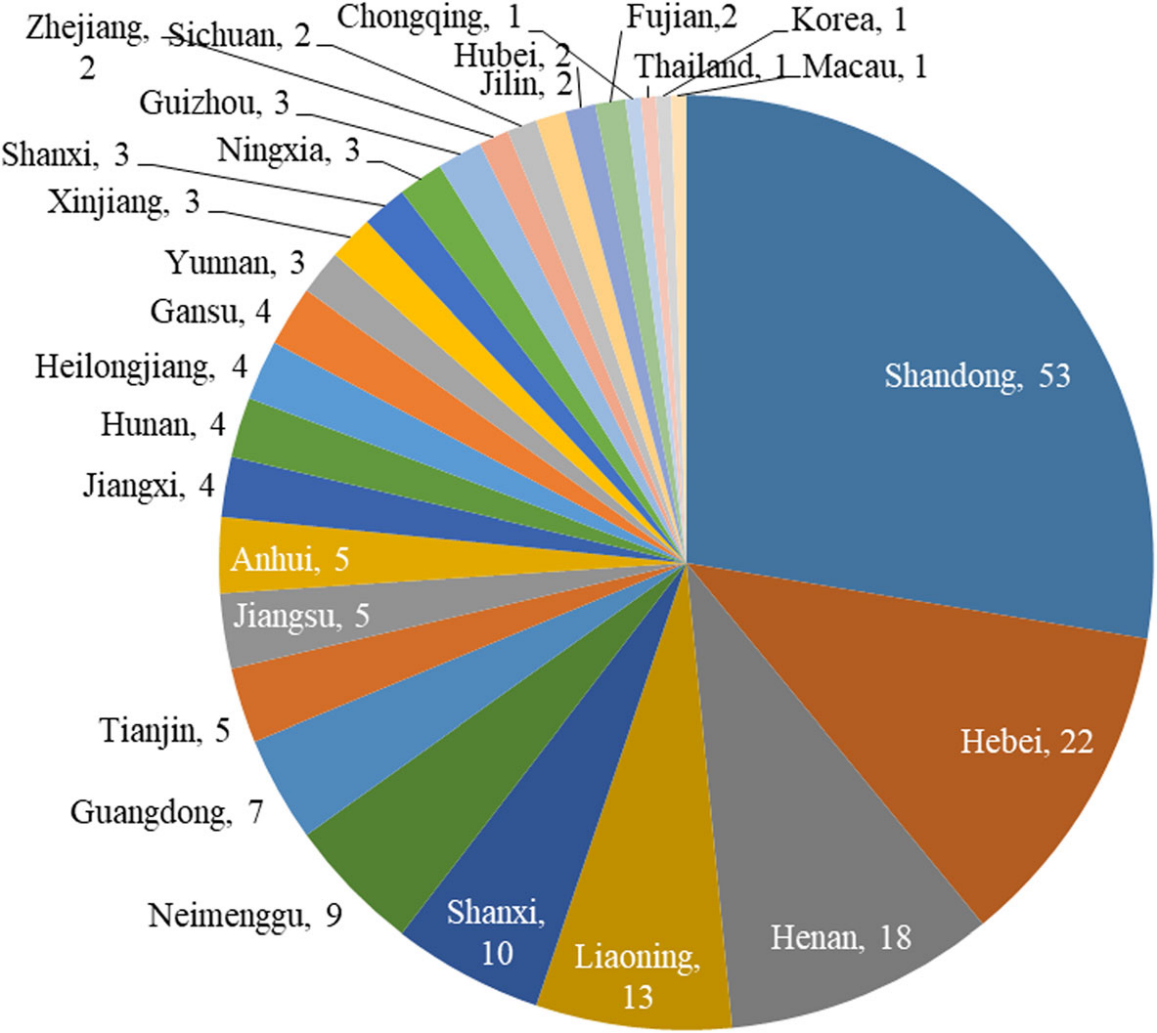
The distribution of 192 PUTH postgraduate medical students outside Beijing (as January 26, 2020)

**Fig. 3:**
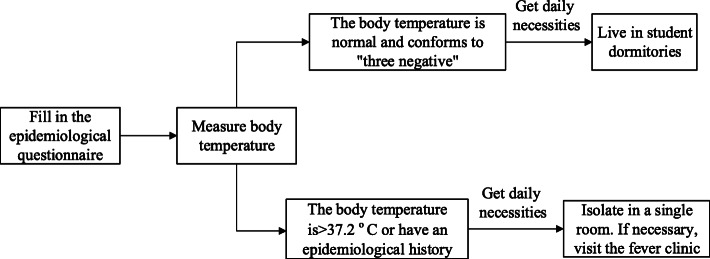
Flowchart of the management of PUTH postgraduate medical students returning to school

**Fig. 4:**
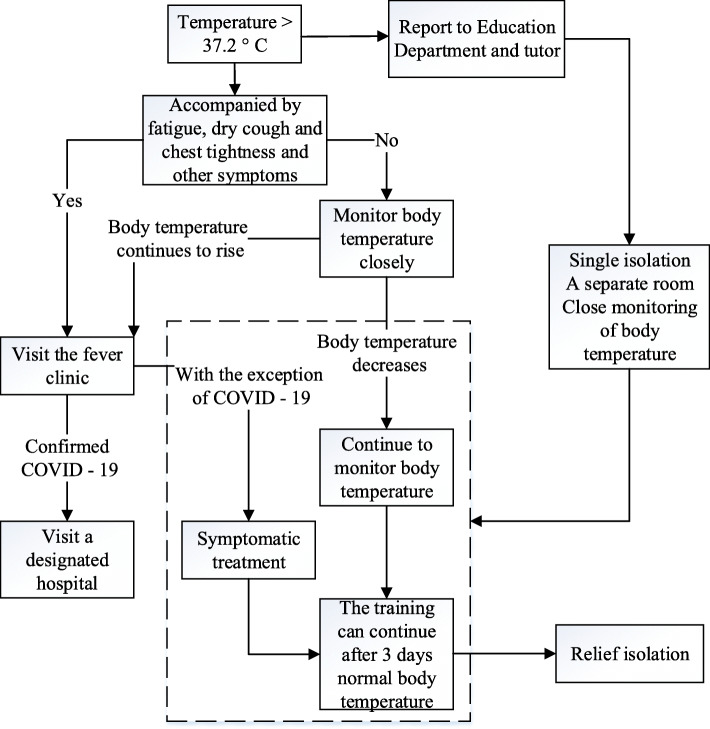
Flowchart of the emergency treatment of students with a fever in PUTH

Considering that postgraduate medical students undertake frontline clinical work, PKUHSC and the hospital decided to implement the principle of allowing graduate students to return in batches with the following considerations: (1) students who had not left Beijing must strictly abide by the clinical rotation arrangements of the hospital, (2) students who had returned to Hubei or passed through Hubei would not return to school, and (3) students returning to other areas, those with no history of contact with confirmed or suspicious cases and those with no fever or respiratory symptoms could return to school. Figure 2 shows the distribution of students on January 26, 2020. To ensure the timely placement of postgraduate medical students returning to school, the Hospital Education Department adopted a standardized flow chart to implement the specifics of various management tasks. Figure 3 shows the flowchart for the management of students returning to school. Figure 4 is a flowchart for the emergency treatment of students with a fever.

#### Reasonable arrangement of clinical work during pandemic prevention and control

According to the “Opinions on Deepening the Cultivation of Clinical Medicine Talents of Medical Education Collaboration”, issued by the Ministry of Education of China, and the “Opinions of the State Council General Office on Further Promoting Medical Education Reform and Development”, issued by the General Office of the State Council, it is necessary to promote master’s degree education as organically connected with the standardized training of residents [5, 6]. Therefore, postgraduate students in clinical medicine degree must complete clinical training in strict accordance with the requirements of the professional training programmes of various disciplines. As the hospital had sent three batches of medical teams to support hospitals in Wuhan, there was a shortage of residents in the emergency department, and more than a dozen postgraduate medical students were temporarily rotated to the emergency department to ensure medical coverage. The clinical work of other departments has since been readjusted, as have the rotation plan and duty positions, according to the specific workload.

#### Laboratory management during the pandemic

**Fig. 5:**
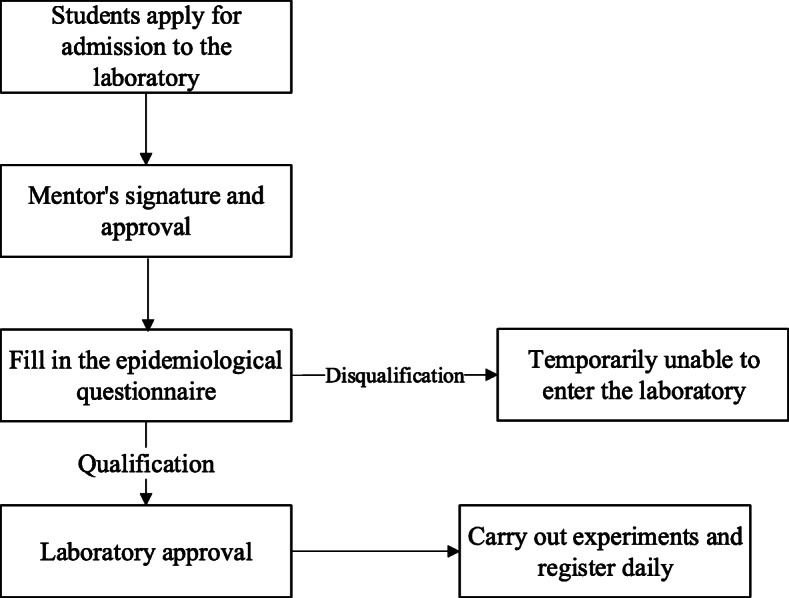
Flow chart for students to enter the laboratory

To allow postgraduate medical students to carry out orderly experiments during the epidemic, the hospital formulated a flow chart for students when entering the laboratory to ensure orderly laboratory work (Fig. 5). During laboratory experiments, postgraduate students were required to abide strictly by the relevant regulations on laboratory safety and protection, implement a daily registration system, and strengthen laboratory management during pandemic prevention and control.

### Teaching and supervision

#### Online teaching was utilized to ensure the quality of teaching for postgraduate students

On February 5, 2020, the Ministry of Education of China issued the “Guiding Opinions on Doing a Good Job in the Organization and Management of Online Teaching in Ordinary Colleges and Universities During the Pandemic Prevention and Control Period”, requiring that online teaching during the pandemic be guaranteed [7]. On February 5, PKUHSC issued the “Pedagogical University’s 2020 Spring Semester Pandemic Prevention and Control Teaching Implementation Plan” to promote the reform of learning methods through innovations of teaching and learning that integrated information technology and education. In accordance with the requirements of the PKUHSC documents, the hospital actively organized teachers to undertake teaching tasks, strengthen the construction of online courses during the pandemic, and adopt online teaching in the form of both recorded and live courses.

#### Strengthen student pandemic prevention and control training

In accordance with the arrangements of the Beijing Municipal Health Commission, the hospital has kept pace with the times, strengthened the training of students in pandemic prevention and control, and organized the creation and revision of training materials from the first to seventh editions. The hospital has simultaneously conducted online and offline training. The training content includes the COVID-19 prevention and control management plan and workflow of PUTH, COVID-19 diagnosis and treatment, and prevention and control principles. Through online theoretical and on-site operation assessment, postgraduate students can fully grasp the COVID-19 diagnosis and treatment plan and hospital infection prevention and control knowledge.

#### Supervisor guidance

A management support team was established to survey the frequency and method of supervisor guidance and to provide real-time advice and support to ensure smooth communication between supervisors and postgraduate students.

#### Dissertation quality management

Requirements for the quality of dissertations have not been reduced by the outbreak. For postgraduate students nearing graduation, the hospital started prereviewing theses in the hospital in early February 2020 and invited experts in various fields to review them through e-mail and other forms. The review content included the thesis content, writing standards, and statistical methods.

### Mental health monitoring

The psychological changes in students during the COVID-19 pandemic were monitored by specialists. During this period, special attention was paid to the psychological changes demonstrated by students, especially those nearing graduation and facing multiple pressures such as the anonymous review of dissertations, thesis defence, employment and stage assessment. The hospital set up a dedicated psychological prevention and control team for students and found that students with psychological problems needed prompt guidance.

### Check

#### Management guidelines

##### Postgraduate temperature monitoring

Full-time teachers of the Education Department monitored the temperature of postgraduate students daily. From January 27, 2020, to March 27, 2020, a total of seven postgraduate students in the hospital developed a fever. The emergency management process for students with fever was strictly followed, and isolation measures were immediately adopted. Among these students, three suffered from fever accompanied by fatigue, dry cough, or chest tightness. Novel coronavirus nucleic acid tests were negative. The other four postgraduate students had only transient low fevers. After symptomatic treatment, their body temperature returned to normal, and normal training resumed after 3 days of normal body temperature. As of May 31, 2020, no postgraduate students had COVID-19.

##### Training of postgraduate medical students returning to the hospital

**Fig. 6:**
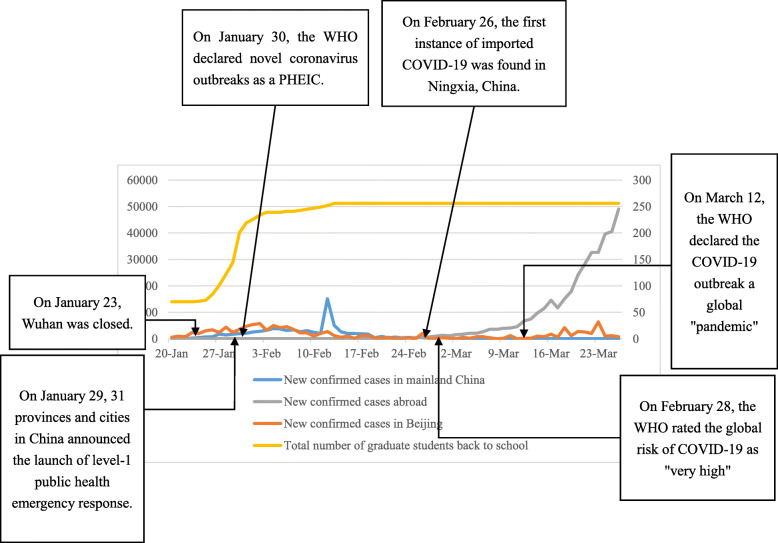
The chart of daily newly confirmed COVID-19 cases worldwide and the return of graduate students in PUTH (information on COVID-19 cases is available from the National Health Commission website)

In late January 2020, after the closure of Wuhan, the hospital urgently arranged temporary dormitories for postgraduate medical students. As of January 31, 2020, the overall return rate of students to the hospital was 79.3%. As of February 14, 2020, the overall return rate of students reached 92.8%. At the beginning of the outbreak, nationwide, most of the students who had left Beijing had already returned to the hospital for clinical work. All students returning to the hospital adopted a centralized management approach for limited regional isolation. In the early stage of the outbreak, professional degree graduates returned to the hospital to participate in clinical work. All returning students were quarantined under centralized management. In the early stage of the outbreak (late January to early February), limited areas were quarantined. During the middle of the outbreak (from early February to mid-February), a strict 14-day quarantine was put into effect for each room (Fig. 6).

##### Clinical rotation

A total of 256 clinical professional postgraduates returned to their resident rotation by the end of March. Almost all rotation departments were covered, although the number of other diseases and cases has declined as a result of the COVID-19 pandemic.

##### Laboratory management

Some students’ experiments were called off due to interruptions to the supply of experimental materials, including reagents and animal models. Fifty-six postgraduates entered the laboratory to continue their experiments. Zero infections occurred in the laboratory during the COVID-19 pandemic.

#### Teaching and supervision

##### Online course teaching effects

Since February 17, 2020, when the hospital completed the construction work for the online teaching of graduate courses, PUTH has implemented online teaching via recorded and live courses, seminars, and other teaching methods. The eight postgraduate courses led by the hospital are being taught as scheduled. To ensure the quality of teaching, the preparation and uploading of each course’s materials have been designated to the person in charge of the course. Questions that arise during the process should be reported to the teacher in time to improve the quality of online teaching. At present, the effects of graduate student feedback regarding online learning are essentially the same as those for offline classroom teaching.

##### COVID-19 prevention and control training effects

On February 6, 2020, postgraduate medical students participated in online training on COVID-19 prevention and control and completed an online assessment. The specific assessment situation is presented in Table 1, which shows that the average score of postgraduate students was relatively low. We conducted intensive training on the weak links revealed through the online assessment, such as the route of transmission, precautions when coughing and sneezing, the disposal of discarded masks, and how to improve immunity. Through such targeted and intensive training, postgraduate medical students have fully mastered the knowledge of pandemic prevention, and the final assessment pass rate was 100%.

**Table 1:** The statistical table of graduate students’ online assessment in PUTH

Graduate student type	Number	Number of respondents	Answered questions per capita	Average score
Professional master’s degree	180	358	1.99	84.65
Professional doctoral degree	96	207	2.16	87.52

##### Survey of supervisor guidance for students

On February 27, 2020, an online questionnaire survey was completed by 84 postgraduate students. The main content was the frequency and method of supervisor guidance. The effective questionnaire recovery rate is 100%. The feedback from the postgraduate students suggested that the supervisor guided them two or more times per week or once per week, accounting for 50.0% (42/84) and 39.3% (33/84) of the sample, respectively. The method of supervisor guidance varies but primarily takes place through WeChat (graphic message), telephone, online voice or video chat and e-mail.

##### Thesis completion and awarding of degrees

As of March 27, 2020, the submission rate of academic dissertations by professional degree graduates was 83.9% (47/56). Among these were several graduate students who were affected by the COVID-19 pandemic and needed to add experiments and data before they could complete their thesis. To this end, both schools and hospitals created plans for awarding degrees and planned to add a degree evaluation committee and subcommittee from July to August 2020 to ensure that all students completed their thesis defence and were awarded degrees.

### Mental health monitoring

A survey of postgraduate psychological status was conducted. On February 12, 2020, the hospital conducted an online questionnaire survey on postgraduate psychology-related issues during pandemic prevention and control. A total of 276 postgraduate students were surveyed, and a total of 198 valid questionnaires were recovered, with an effective questionnaire recovery rate of 71.7%. Table 2 shows that 66.4% (77/116) of master’s degree students experienced some anxiety or depression in the face of pandemic-related pressure; 31.9% (37/116) did not feel obvious psychological and mental pressure. A total of 59.8% (49/82) of doctoral degree students experienced some anxiety or depression, while 40.2% (33/82) did not feel obvious psychological and mental pressure. The difference between the two types of postgraduate students, *P* > 0.05, is not statistically significant. The specific results are shown in Table 2. However, compared with doctoral students, master’s degree students were more prone to anxiety or depression. This result shows that doctoral students have more clinical experience in the face of pandemics and are more rational in the face of emergency situations. These master’s degree students are more prone to psychological problems such as tension and anxiety, which suggests that the teaching management department, clinical departments and supervisors should pay more attention and provide key guidance to these postgraduates.

**Table 2:** Survey table of psychological status of postgraduates in PUTH [*n* (%)]

Question	Options	Professional master’s degree (*n* = 116)	Professional doctoral degree (*n* = 82)	χ^2^	*P*
Reacting to the pandemic, I self-assessed my mental state in the past 7 days	Do not feel obvious psychological and mental pressure	37 (31.9)	33 (40.2)	2.692	0.260
	Have some anxiety or depression, can bear it and adjust myself, and can now gradually adapt and improve	77 (66.4)	49 (59.8)		
	Very anxious or depressed, feelings do not alleviate over time, seriously affects normal study and work	2 (1.7)	0		

### Act

#### Management guidelines

Thoughtful and responsive management guidelines are essential under the pressure of the pandemic. In addition to the daily monitoring of body temperature, completion of online teaching, and guarantee of thesis quality, the managers of postgraduate students adopted multiform fine management methods such as simulation teaching, project-based learning (PBL) teaching, case-based learning (CBL) teaching, and video conferencing to provide training programmes. The next stage will focus on enhancing the diversity of clinical training methods for postgraduate students. In addition, those postgraduate students who are still unable to return to the hospital or whose research has been affected by the pandemic can use this time to strengthen their knowledge of academic frontiers and cross-disciplinary literature, data statistics, etc., through tools such as the cloud experiment platform to strengthen their experimental operational skills.

#### Teaching and supervision

##### Increase the content of training courses

During the COVID-19 pandemic, it was found that postgraduates have less training in public health and knowledge of infectious disease prevention and control. Although most of the students have taken public health and infectious-disease-related courses at the undergraduate level, this field of knowledge is rapidly updated, and postgraduate students need to be trained in the normalization of maintaining public health and infectious disease prevention and control knowledge. Graduate students of all majors need to have a deep understanding of the principles of handling public health emergencies and the protection requirements for infectious diseases.

##### Increase the clinical training for infectious diseases

According to the requirements of the current postgraduate training programme, only internal medicine majors can participate in 2 months of clinical training for infectious diseases, whereas students in other majors have no opportunity to participate in the practice of infectious disease. To better cope with emergency prevention and control work related to infectious diseases in the future, it is recommended that flexible time in other professional graduate training programmes be used for clinical training for infectious diseases. The duration of such flexible time is approximately 2 weeks, which is sufficient time to allow for postgraduate students to master the clinical treatment of common infectious diseases.

#### Mental health support is necessary

Medical postgraduate students are already facing considerable clinical and scientific pressure, and the outbreak of the epidemic has increased their psychological burden. Effective support should be provided to help them cope with these pressures. Student mental health profiles are created and followed up by professional teams. The COVID-19 pandemic urges us to improve and professionalize our psychological monitoring of students and provide sustained and sufficient attention to the mental health of medical postgraduate students.

## Discussion

**Fig. 7:**
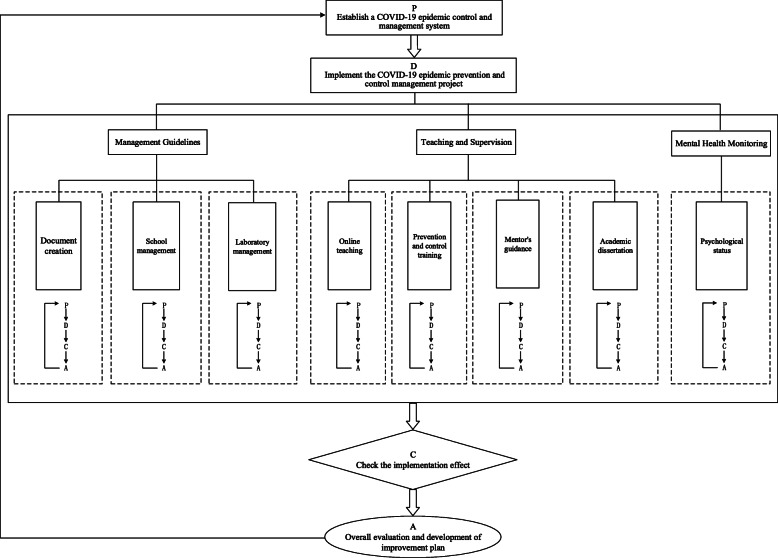
Management model for clinical medicine graduate students in PUTH during the epidemic

The COVID-19 pandemic has exerted a massive impact on medical education, including daily management, teaching methods [8], clinical practice [9] and mental health support [10]. This study finds that PDCA is an effective management tool for the management of medical postgraduate students during public health emergencies. Through the implementation of this round of the PDCA cycle, a management model map for postgraduates under a special pandemic situation was formed. See Fig. 7 for details.

Management departments first need to perform comprehensive and detailed consideration of all possible impacts of the epidemic and then to put forward targeted plans to ensure the orderly development of clinical and scientific research training for medical postgraduates. The closed-loop management strategy of PDCA could help managers achieve high management quality and deal with public health emergencies.

In the future, we will continue to improve the content of the four stages of PDCA according to the management model map and continuously improve the quality of teaching management and management efficiency regarding public health emergencies.

## Conclusions

PDCA cycles play an effective role in the management of medical postgraduates in the COVID-19 pandemic prevention and control. The Education Department of PUTH used PDCA management strategies and innovative work methods to form an management system for the linkage of the hospital, clinical department, supervisor, and postgraduate students and further used information technology to strengthen the daily management of postgraduate students, academic guidance, and online teaching, ensure the quality of medical talent cultivation, and attend to both the physical and mental health of postgraduate students.

## Supplementary Information

**Additional file 1: Supplementary file 1.** Questionnaire Survey.

## Data Availability

All data generated or analysed during this study are included in this published article. A license was required to use any of the data collection instruments in this study upon reasonable request and with permission of the authors.

## Abbreviations

PUTH: Peking University Third Hospital
PDCA: Plan-do-check-act
WHO: World Health Organization
PHEIC: Public health emergency of international concern
PDS: Plan-do-see
PKUHSC: Peking University Health Science Centre
