# Supplementary material for: Application of PDCA cycle management for postgraduate medical students during the COVID-19 pandemic
Source: BMC Med Educ. 2021 May 29;21:308. doi: 10.1186/s12909-021-02740-6 (PMC8164051; doi:10.1186/s12909-021-02740-6)
Supplement: Supplementary file 1 — Additional file 1: Supplementary file 1. Questionnaire Survey. [file 12909_2021_2740_MOESM1_ESM.docx]

**Supplementary File 1**

Questionnaire Survey

Hello, everyone! To further understand your current psychological situation, we have specially designed this questionnaire. If you agree to complete this questionnaire, please answer the following questions. The survey is anonymous. All data are for statistical analysis only. Please feel free to fill in. Thank you for your support!

Question

1. Reacting to the epidemic, I self-assessed my mental state in the past 7 days.

a. Do not feel obvious psychological and mental pressure

b. Have some anxiety or depression, can bear it and adjust myself, and can now gradually adapt and improve

c. Very anxious or depressed, feelings do not alleviate over time, seriously affects the normal study and work

Hello, everyone! To understand the situation of tutor guidance of graduate students, we have specially designed this questionnaire. If you agree to complete this questionnaire, please answer the following questions. The survey is anonymous. All data are for statistical analysis only. Please feel free to fill in. Thank you for your support!

Question

1. The frequency of tutor guiding the graduate students

a. More than twice a week

b. Once a week

c. Less than once a week

2. What are the methods of tutor guidance? (multiple choice)

a. WeChat (graphic information)

b. Telephone

c. Online voice

d. Video chat

e. E-mail
